# Acute septic thrombophlebitis of the lower extremities due to foreign body injury and infection: a case report

**DOI:** 10.1186/s12879-023-08245-5

**Published:** 2023-05-02

**Authors:** Guofu Zheng, Daoxiong Xiao, Hailiang Xie, Minggui Lai, Bo Ye, Xiaochun Liu

**Affiliations:** 1 The Department of Vascular & Hernia Surgery, Ganzhou People’s Hospital, Ganzhou, 341000 Jiangxi Province China; 2 The Department of Medical Imaging, Ganzhou People’s Hospital, Ganzhou, 341000 Jiangxi Province China

**Keywords:** Foreign body, Scrub typhus, Septic thrombophlebitis, Injury, Case report

## Abstract

**Background:**

Septic thrombophlebitis (STP) of the lower extremities caused by foreign bodies is rare in the clinic, and the symptoms are serious. If the correct treatment is not implemented as soon as possible, the patient may progress to sepsis.

**Case presentation:**

We report the case of a 51-year-old normally healthy male who developed fever 3 days after field work. When he was weeding with a lawn mower in the field, a metal foreign body from the grass flew into his left lower abdomen, resulting in an eschar on his left lower abdomen. He was diagnosed with scrub typhus but did not respond well to anti-infective treatment. After a detailed inquiry of his medical history and an auxiliary examination, the diagnosis was confirmed as STP of the left lower limb caused by a foreign body. After surgery, anticoagulation and anti-infection treatment, the infection and thrombosis were controlled, and the patient was cured and discharged.

**Conclusions:**

STP caused by foreign bodies is rare. Early detection of the aetiology of sepsis and early adoption of the correct measures can effectively block the progression of the disease and reduce the patient’s pain. Clinicians should identify the source of sepsis through a medical history and clinical examination.

## Background

Deep vein thrombosis (DVT) of the lower extremities is a common disease in hospitalized patients in the vascular surgery department. However, Septic thrombophlebitis (STP) is relatively rare in the clinic. Metal foreign bodies with a large number of bacteria can lead to vein injury and infection [1]. STP caused by the continuous migration of foreign bodies that enter the body due to trauma is uncommon.

In this case, the patient presented with sepsis because of an eschar on the body surface and was in the epidemic area of scrub typhus [2], and this type of case could be extremely easily misdiagnosed as scrub typhus in the early stage, leading to confusion in the diagnosis and treatment.

## Case presentation

**Fig. 1:**
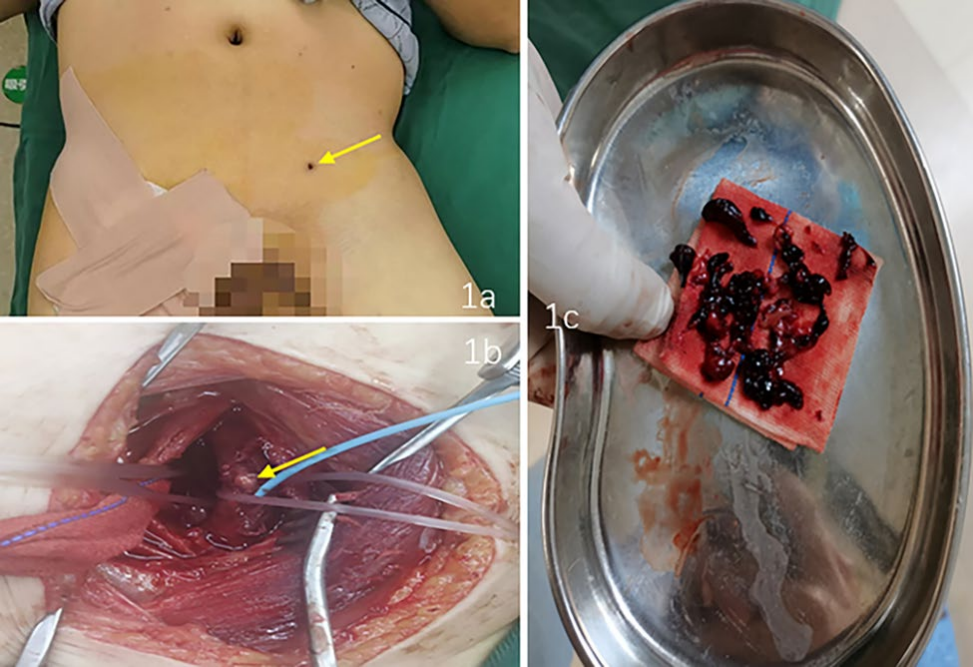
There appeared to be an eschar on the lower left abdomen (1a: arrow). A Fogarty balloon catheter was used for thrombectomy (1b: arrow). A mixed septic thrombus approximately 16 cm in length was removed(1c)

**Fig. 2:**
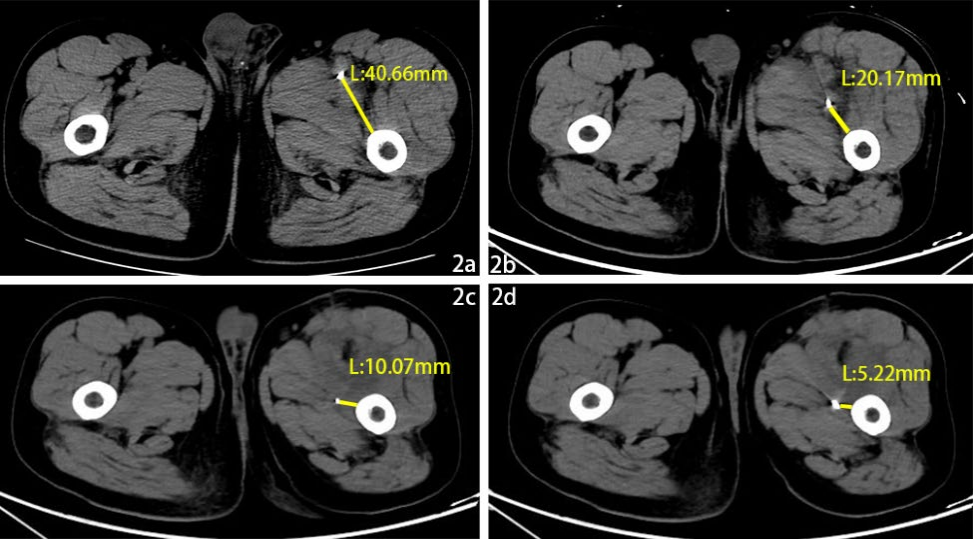
The distance between the metal foreign body and the femur at different times

**Fig. 3:**
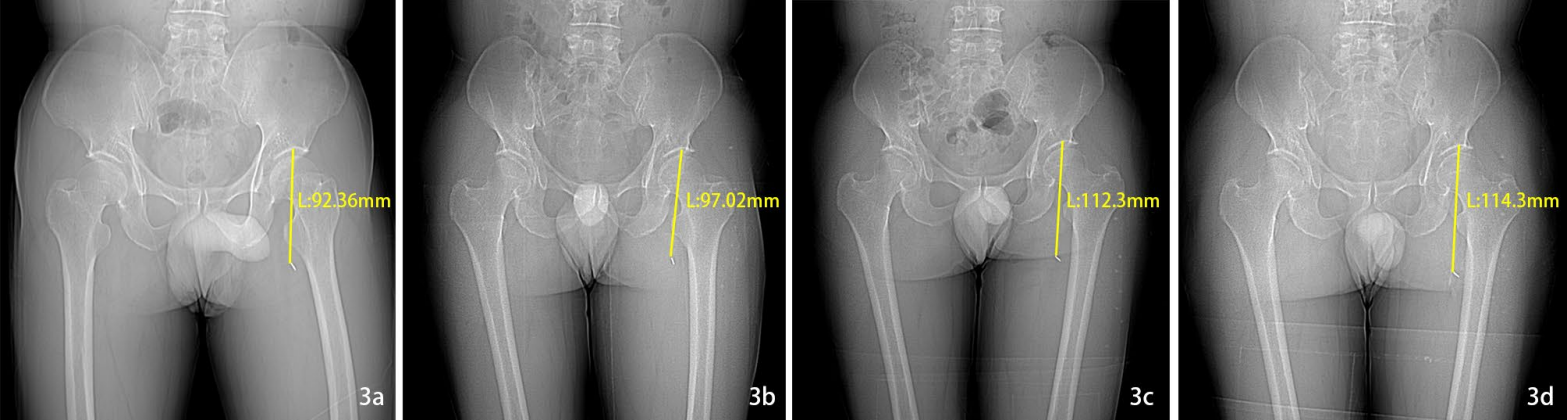
The X-ray showed the different location of the metal foreign body at different times (anteroposterior position)

**Fig. 4:**
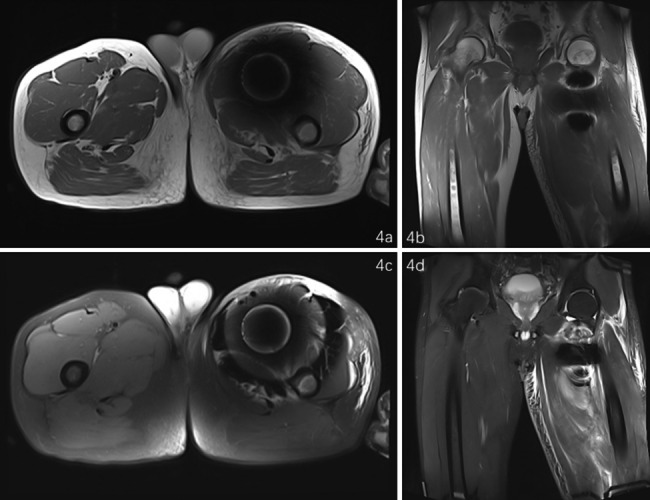
MRI showed metal artefact images of the thigh in different MRI sequences (a, b as T1 sequences and c, d as T2 sequences)

A 51-year-old male had worked in the field with a lawn mower, and ten days later, there appeared to be an eschar due to an insect bite on the lower left abdomen (arrow, Fig. 1a). The patient went to the local hospital, and scrub typhus was considered. After 4 days of anti-infective treatment with doxycycline, the patient continued to have chills and fevers and then gradually developed swelling and pain in the left lower extremity. Four days later, he was transferred to the emergency department of our hospital. This patient had been in good health previously. On admission, his body temperature was 39.8 °C, pulse rate was 115 beats/min, respiratory rate was 25 breaths per minute, his SpO2 was 100% while he was receiving supplemental oxygen and blood pressure was120/83 mmHg. His abdomen was soft without tenderness. The laboratory results were as follows: white blood cell (WBC), 39.32*10^9^/L; D-dimer, 7.06 mg/L; and C-reactive protein, 67.45 mg/L. Computed tomography (CT) and X-ray examination of the abdomen revealed no significant abnormalities, but a metal foreign body was visible in the left thigh root (Figs. 2a and 3a). The patient’s left lower limb swelling and pain continued to worsen, but no attention was given. The patient was treated with moxifloxacin and cefoperazone sodium sulbactam sodium for anti-infection according to the diagnosis of scrub typhus. After three days, blood culture results showed the presence of bacteraemia. The matrix-assisted laser desorption ionization time-of-flight mass spectrometry (MALDI-TOF MS) was used to confirm that the pathogen was *Escherichia coli*. And the result of the Weil–Felix agglutination test was negative. Further computed tomography angiography (CTA) examination of the lower limbs revealed soft tissue swelling and DVT in the left thigh, including gas in the common femoral vein and a metal artefact adjacent to the deep femoral artery. The metallic foreign body was significantly displaced downwards and backwards in the thigh, and it had gradually approached the femur. (Figures 2b and 3b). However, at that time, the clinician did not read the CT images carefully and was unware of the existence of the metal body in the thigh. Magnetic resonance (MR) further confirmed muscle oedema, effusion infection, DVT, and a metal artefact adjacent to the deep femoral artery (4a, 4b as T1 sequences and 4c, 4d as T2 sequences, Fig. 4). Further examination of the patient’s medical history revealed that when he was weeding with a lawn mower in the field three days before his fever, a metal foreign body from the grass flew into his left lower abdomen, resulting in an eschar on his left lower abdomen (Fig. 1a). At that time, the diagnosis became clear: STP and sepsis caused by a metal foreign body injected into the body from trauma. Due to the expansion and contraction activities of muscles, the foreign body migrated down the deep femoral vein space, which might cause further damage to the femoral vein and produce more thrombosis.

On the fifth day of hospitalization, the patient was transferred to the vascular surgery department, and surgery was planned. The vena cava filter was placed on the same day, followed by X-ray positioning with a C-arm machine under general anaesthesia intubation. A longitudinal incision was made on the medial side of the left thigh root. The muscle space was gradually dissociated, and oedema and adhesion of the surrounding tissues and inflammatory secretions around the femoral vein were found. The local vein wall was white. The femoral vein was dissected, the contents were found to be pus, and 1 ml was aspirated for culture (result: *Escherichia coli*). A Fogarty balloon catheter was used for thrombectomy, and a mixed septic thrombus approximately 16 cm in length was removed (Fig. 1b and c), but the metal foreign body could not be found. The vena cava filter was removed the next day. Imipenem cilastatin sodium was used to strengthen the anti-infection treatment, and anticoagulant therapy was continued. The patient’s WBCs gradually decreased to 7.33*10^9^/L, and he was discharged 2 weeks after surgery. He continued to receive anticoagulant therapy and wore elastic socks on his lower limbs at home. During the six-month follow-up period, he had no fever, swelling or pain in the left lower extremity, and ultrasound showed that the femoral vein thrombosis was mostly absorbed. However, the metal foreign body continued to migrate deep into the medial side of the femur (Figs. 2c and d and 3c and d) but did not cause further injury or infection. To date, the patient has been followed up for ten months and is in a stable condition.

## Discussion

Retained foreign bodies might lead to sepsis [3, 4]. However, STP caused by foreign bodies has rarely been reported. In the previous reports, STP occurred in the internal jugular vein and inferior vena cava, and the retained foreign bodies were a needle and a toothpick, respectively [1, 3]. There were occasional reports of foreign bodies in the thigh, such as glass[5] and wood[6], but these foreign bodies did not cause infection and thrombosis to the patients.

Scrub typhus, which is an acute febrile illness caused by *Orientia tsutsugamushi*, is a serious public health problem in the Asia-Pacific area [7]. The diagnosis of scrub typhus is based on the patient’s history of exposure, clinical features, and results of serologic testing. An eschar at the wound site is the single most useful diagnostic clue [8]. A positive Weil–Felix test result can also confirm the diagnosis [8]. Most patients have a history of field work, and the incubation period is 5–14 days. The onset of the disease is acute, with high fever, toxaemia, rash, eschar, lymphadenopathy and other characteristic clinical manifestations [7].

This patient’s clinical presentation of high fever, sepsis, history of field work, characteristic left lower abdominal skin surface eschar, response to antibiotics, and endemic area of scrub typhus [2] made his early diagnosis of scrub typhus seem reasonable. However, the patient had no clinical manifestations in the lungs. The eschar was a skin rupture caused by a metal foreign body entering the body, and the negative result of the Weil–Felix agglutination test together with the imaging of the infected lesion in the left thigh root and the artefact of the metal foreign body helped rule out the diagnosis of scrub typhus.

Other reasons for misdiagnosis include not taking a careful medical history or having a preconceived diagnosis (based on one’s own experience). In addition, the patient did not provide timely information about the injury while working in the field, which also made misdiagnosis possible.

At present, iatrogenic factors related to venous catheter infection are the main causes of STP [9, 10]. Various central venous catheters and haemodialysis tubes are placed in vessels for a long time due to improper nursing care or decreased resistance to infection, resulting in the infection of venous catheters and then the formation of iatrogenic septic thrombosis. STP caused by drug abusers repeatedly piercing their own deep veins without strict disinfection has also been reported [11]. STP of the inferior vena cava resulting from an ingested foreign body is rare [12]. STP resulting from injury to the deep veins by acupuncture has also been reported [1, 13].

However, in this patient, a metal foreign body with a large number of bacteria entered the body due to trauma, and the foreign body constantly migrated. The local and systemic symptoms of the patient changed over time, from mild symptoms in the early stage to severe local and systemic symptoms of infection in the later stage. Initially, the foreign body stayed in the superficial muscular layer. At that time, open surgery was performed as soon as possible to find the foreign body to prevent further migration and infection of the femoral vein, leading to septic thrombosis. Because the foreign body was shallow and the local oedema was not serious at that point, the chance of finding the foreign body appeared better.

There are different methods for the management of septic thrombosis, including conservative treatment [10], interventional thrombectomy [9, 12], and open thrombectomy. In addition to STP, the patient had severe infection in the surrounding tissues, and bacteria was cultured from the blood. After 10 days of intravenous antibiotics, the infection could not be controlled, and the patient developed sepsis. The foreign body should also be removed if possible. Therefore, open surgery was performed to remove the pus embolus and drain the lesion. To prevent the formation of pulmonary embolism and metastatic abscess due to the discharge of septic thrombus, a filter was placed into the inferior vena cava prior to surgery. Because of bacteraemia, which could lead to septic thrombosis in the colander, we removed the filter the day after thrombectomy.

In addition to local treatment of infectious lesions, anti-infection treatment is very important for sepsis. After the surgery, more appropriate antibiotics were prescribed, which accelerated the killing of bacillus in the blood. The patient’s blood culture result was negative one week after surgery. Due to the infection, the patient developed anaemia and hypoproteinaemia. Transfusion of blood products such as red blood cells, plasma, and albumin is also indispensable to improve these conditions and enhance the patient’s ability to fight infections.

Ideally, the foreign body should be removed. However, the foreign body was too small to be removed in this case. At the time, the operation had lasted 6 h, and the local and systemic infection was severe. Therefore, we had stop trying to remove the foreign body from the tissue in the first stage. *Escherichia coli* in the tissues was destroyed by local and systemic treatment, and the retained foreign body was then less likely to reinfect the body. However, due to the expansion and contraction activities of muscles, foreign bodies may migrate, which may further damage local tissues, especially nerve and vascular tissues; due to the expansion and contraction activities of muscles, the foreign body migrated down the deep femoral vein space, which may cause further damage to the femoral vein and produce more thrombosis. therefore, long-term follow-up is needed. If necessary, the foreign body can be removed in the second stage under the guidance of digital subtraction angiography (DSA). If the foreign body stops migrating due to local scarring or adhesion and there is no harm to the body, there is no need to surgically remove the foreign body [14].

In conclusion, STP caused by foreign bodies is rare. For patients with sepsis after foreign bodies enter the body who gradually develop swelling and pain in the lower extremities, a detailed medical history should be reviewed, the possibility of the formation of STP should be considered as early as possible, and the diagnosis should be corrected. Then, the appropriate measures can be taken earlier, which may lead to better clinical outcomes.

## Data Availability

Not applicable.

## Abbreviations

STP: septic thrombophlebitis
DVT: deep vein thrombosis
WBC: white blood cell
CT: computed tomography
MALDI-TOF MS: matrix-assisted laser desorption ionization time-of-flight mass spectrometry
CTA: computed tomography angiography
MR: magnetic resonance
DSA: digital subtraction angiography.
